# A flexible MRF approach to improve kinetic rate estimation with bSSFP‐based hyperpolarized [1‐ ^13^C]pyruvate MRI

**DOI:** 10.1002/mrm.30466

**Published:** 2025-03-04

**Authors:** Anna Bennett Haller, Xiaoxi Liu, Avantika Sinha, Sule Sahin, Peder E. Z. Larson, Charlie Yi Wang

**Affiliations:** ^1^ UC Berkeley–UCSF Graduate Program in Bioengineering University of California, Berkeley and University of California, San Francisco California USA; ^2^ Radiology and Biomedical Imaging University of California San Francisco California USA

**Keywords:** ^13^C, bSSFP, hyperpolarized, lactate, metabolic imaging, MRF, MRI, pyruvate

## Abstract

**Purpose:**

In this work, we adopt the MR fingerprinting (MRF) framework and leverage its flexibility in quantitative pulse sequence design to propose improved balanced steady‐state free precession (bSSFP)–based hyperpolarized Carbon‐13 (^13^C) acquisitions for robust metabolic conversion rate quantification.

**Methods:**

Spectrally selective bSSFP‐based acquisitions with variable RF excitation were implemented for [1‐^13^C]pyruvate and used in conjunction with prior implementation of [1‐^13^C]lactate selective bSSFP imaging. MRF framework parameter estimation was performed using dictionary‐based template matching. Influences of bSSFP‐based acquisitions and sigmoid RF excitation scheme were assessed with simulation experiments and Monte Carlo evaluation. Methods were then compared using experimental data from rat kidney acquired on a clinical 3 T scanner.

**Results:**

Simulations indicated that combining bSSFP‐based acquisitions and variable RF excitation (MRF‐Sigmoid) exhibited bias <0.1% across the majority (86%) of combinations of pyruvate‐to‐lactate conversion rate (k_PL_) and noise level investigated when estimating k_PL_ with the MRF framework. bSSFP‐based experiments, with and without sigmoid excitation scheme, showed lower variance in fits at all levels of k_PL_ and noise investigated compared to the method used in prior work by this group (hybrid gradient echo). Positive, linear correlations were found for in vivo voxel‐wise estimates of k_PL_ in healthy rat kidneys when comparing all experiment methods. MRF‐Sigmoid experiment design increased pyruvate cumulative SNR by 3.5‐fold over hybrid gradient echo while maintaining similar lactate cumulative SNR.

**Conclusion:**

The use of the MRF framework for k_PL_ estimation demonstrates the feasibility of dictionary‐based template matching and can be used to accurately estimate physiologically relevant k_PL_ and improve cumulative SNR.

## 1 INTRODUCTION

Carbon‐13 (^13^C) MRS is a powerful noninvasive tool for the study of metabolism. With dissolution dynamic nuclear polarization, labeled hyperpolarized (HP) tracers can be administered with several thousand‐fold increased signal. However, the low physiologic concentration of these agents, and their irreversible T_1_ dependent relaxation, result in sensitivity and resolution limitations, and their use remains challenging. Therefore, robust and efficient acquisition schemes must be employed to maximize the information obtained.

Many HP ^13^C methods have been explored, attempting to balance the competing needs of high spatiotemporal resolution and sensitivity, including MRSI‐, CSI‐, and EPI‐based imaging. Metabolite‐specific imaging techniques in particular are fast, RF‐efficient, and well suited for [1‐^13^C]pyruvate imaging.^1^ Additionally, they are readily combined with other sequence designs to further accelerate acquisition, improve robustness to field inhomogeneity, or increase efficiency. Notably, balanced steady‐state free precession (bSSFP) is particularly attractive due to its high theoretical SNR per unit time. In recent years, several bSSFP‐based implementations of HP ^13^C MR have been developed to improve SNR and sensitivity, including for pyruvate‐lactate exchange,^2^, ^3^, ^4^ with results suggesting two‐ to threefold increased signal SNR over metabolite‐specific gradient echo (GRE) methods in both animal and human imaging studies. However, in the case of [1‐^13^C]pyruvate, quantification of metabolic conversion in HP ^13^C MRI—specifically pyruvate‐to‐lactate conversion rate (k_PL_)—remains a key ingredient for clinical application and interpretation, and bSSFP‐based ^13^C modeling and quantification for metabolic rates has only recently started to be explored.^5^ Additionally, whereas variable flip‐angle design approaches have been found to potentially increase SNR in CSI, EPSI, and bSSFP methods,^6^, ^7^, ^8^, ^9^, ^10^ the benefits have not been extrapolated to bSSFP for HP ^13^C methods.

MR fingerprinting (MRF) is a generalized framework for pulse sequence design and reconstruction, particularly suited for multiparametric quantitative MR applications. In brief, the framework relies on the principle that, given the underlying signal model of an MR experiment, a dictionary of possible tissue signals can be constructed regardless of sequence design. After data acquisition, this dictionary, combined with a pattern‐matching approach, can be used to identify the most likely underlying tissue properties to generate the observed data. This approach is incredibly versatile with respect to pulse sequence design, and this flexibility was initially shown in multiparametric proton MRI applications^11^ where pseudo‐random acquisition was adopted to better measure T_1_ and T_2_. This framework has since been adapted to a wide array of applications, including MRS.^12^, ^13^, ^14^

In this work, we adopt the MRF framework and leverage its flexibility in quantitative pulse sequence design to propose improved bSSFP‐based HP ^13^C acquisitions for metabolic conversion rate quantification robustness. Whereas the pulse sequence design space is infinite, we also leverage the MRF framework to explore the potential benefit of a variable flip angle bSSFP approach.

## 2 METHODS

### 2.1 Acquisition and sequence design

**FIGURE 1:**
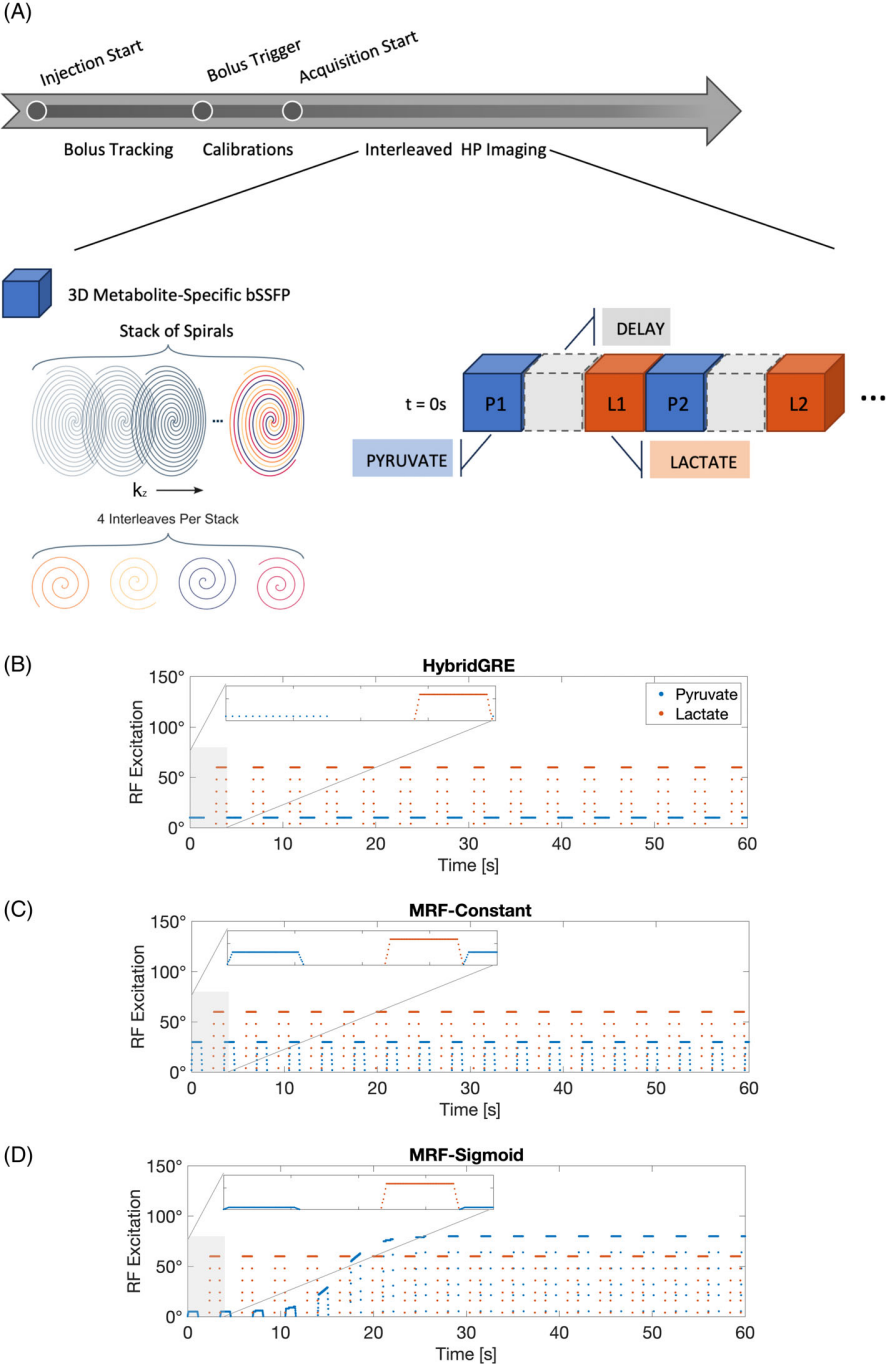
(A) HP imaging experiment design consisted of bolus‐tracking images, initiated at the start of injection, followed by center frequency and B_1_^+^ calibrations prior to running interleaved metabolite‐specific imaging. (B) Prior work (HybridGRE) consisted of interleaved 2D GRE for pyruvate and 3D bSSFP for lactate metabolite‐specific imaging sequences. (C) Proposed method (MRF‐Sigmoid or MRF‐Sigm) with interleaved pyruvate and lactate‐specific 3D bSSFP acquisitions. Additionally, the proposed method varies the RF excitation throughout the full acquisition window, increasing from 5 through 80 degrees following a sigmoid function. bSSFP, balanced steady‐state free precession; GRE, gradient echo; HP, hyperpolarized; k_PL_, pyruvate‐to‐lactate conversion rate; MRF, MR fingerprinting.

The experimental design for investigated pulse sequence designs are shown in Figure 1. Following HP [1‐^13^C]pyruvate administration with bolus tracking and automated calibrations, an interleaved acquisition block structure was implemented. Within a given acquisition block, spectrally selective RF excitation was used to detect a single metabolite, in line with prior implementations developed for [1‐^13^C]lactate, ^13^C‐urea, and ^13^C‐bicarbonate imaging.^2^, ^3^, ^15^, ^16^ For investigational comparison, three pulse sequence designs were investigated in detail in both simulation and in vivo: a combined GRE with bSSFP hybrid approach (HybridGRE), constant flip angle bSSFP (MRF‐Constant), and variable flip angle bSSFP (MRF‐Sigmoid).

HybridGRE (previously referred to as *MS‐3DSSFP* ^*2*^, renamed in this work for clarity) was selected as a starting point for investigation due to previous results demonstrating two‐ to threefold increased SNR over GRE‐only–based approaches, in line with theoretical expected SNR improvements. The acquisition details for all experiments are detailed in Table 1. Briefly, the HybridGRE method used a bSSFP approach within lactate‐sampling acquisition blocks, and a GRE approach in pyruvate‐sampling acquisition blocks, with applied true flip angle at each RF excitation shown in Figure 1B. A spectral–spatial multiband RF pulse model^17^ was used to develop the [1‐^13^C]pyruvate selective GRE excitation with 25 ms pulse duration. The bSSFP acquisition approach for [1‐^13^C]lactate sampling employed a spectrally selective excitation pulse with minimized duration of 9 ms designed from multiband RF pulse model.^18^

**TABLE 1:** Hyperpolarized experiment parameters for Monte Carlo simulations and small animal studies.

Experiment Type	Pyruvate Selective Acquisition	Lactate Selective Acquisition
A MRF‐Sigmoid	3D bSSFP flip = Sigmoid, t_readout_ = 4.2 ms, TR = 15.6 ms, FOV = 8 × 8 × 33.6 cm, temporal resolution 4.1 s, resolution = 2.5 × 2.5 × 21mm	3D bSSFP flip = 60°, t_readout_ = 3.8 ms, TR = 15.3 ms, FOV 8 × 8 × 33.6 cm, resolution = 2.5 × 2.5 × 21mm
B MRF‐Constant	3D bSSFP flip = 30°, t_readout_ = 4.2 ms, TR = 15.6 ms, FOV = 8 × 8 × 33.6 cm, temporal resolution 4.1 s, resolution = 2.5 × 2.5 × 21mm	
C HybridGRE	multi‐slice 2D MS‐GRE flip = 10°, t_readout_ = 22 ms, TR = 1000 ms FOV = 8 × 8 cm, slice thickness = 21 mm, temporal resolution 4.6 s, resolution = 2.5 × 2.5 mm	

Abbreviations: bSSFP, balanced steady‐state free precession; GRE, gradient echo; MRF, MR fingerprinting.

Both MRF‐Constant and MRF‐Sigmoid approaches utilized bSSFP approach for both pyruvate‐ and lactate‐specific imaging. Spectrally selective excitations for [1‐^13^C]pyruvate, newly developed, and [1‐^13^C]lactate, the same as the HybridGRE method, were developed from a multiband RF pulse model.^18^ These excitations had minimized 9 ms duration, and minimal excitation of [1‐^13^C]alanine, [1‐^13^C]pyruvate‐hydrate, and ^13^C‐bicarbonate resonances at 3 T as previously described.^3^, ^15^, ^16^

For constant flip angle bSSFP (MRF‐Constant) and variable flip angle bSSFP (MRF‐Sigmoid) methods, pyruvate acquisition blocks were switched to bSSFP acquisitions. For the pyruvate bSSFP excitations, phase was alternated at 15.6 ms TR to minimize bSSFP banding overlap with off‐resonance metabolites similar to previous methods.^2^, ^3^, ^16^ The MRF‐Sigmoid pyruvate RF flip angle scheme was based on a modified logistic function on a per excitation basis (shown in Figure 1D):

(1) $$\alpha \left(t\right)=\alpha_{\min}+\frac{\alpha_{\max}-\alpha_{\min}}{1+{ℯ}^{\left(20-t\right)}},$$

where $a_{\min}=5^{{}^{\circ}}$ is the minimum/initial RF flip angle, $\alpha_{\max}=80^{{}^{\circ}}$ is the maximum RF flip angle, and t is the elapsed time in seconds after acquisition start. The catalyzation and de‐catalyzation pulses, preparation pulses used to facilitate transitions to and from steady‐state, were determined using the same nonlinear ramp‐up and ramp‐down calculation defined by prior methods.^2^ The series of ramped pulses were calculated relative to the first and last excitation tip angles independently for each time point.

Image data was reconstructed utilizing Kaiser‐Bessel kernel–based gridding (oversampling factor = 1.4, kernel width = 4.5) and the reconstruction pipeline established in prior work.^2^

### 2.2 Fingerprint simulation and dictionary matching

Generation of signal time courses, or fingerprints, were adapted from previous methods^12^ solving the modified Bloch‐McConnell equations with exchanging pools of pyruvate and lactate magnetization using in‐house developed MatLab code (Mathworks, Natick, MA). Each fingerprint required 10 input parameters: pyruvate‐to‐lactate (k_PL_) forward conversion rate, pyruvate and lactate chemical shift frequency, T_1_, and T_2_ (ω_Pyruvate_, T_1,Pyruvate_, T_2,Pyruvate_, ω_Lactate_, T_1,Lactate_, T_2,Lactate_), pyruvate bolus arrival time and bolus duration (T_Bolus Arrival_ and T_Bolus Duration_),^6^ and intravoxel field inhomogeneity characterized by linewidth (LW).^12^

**FIGURE 2:**
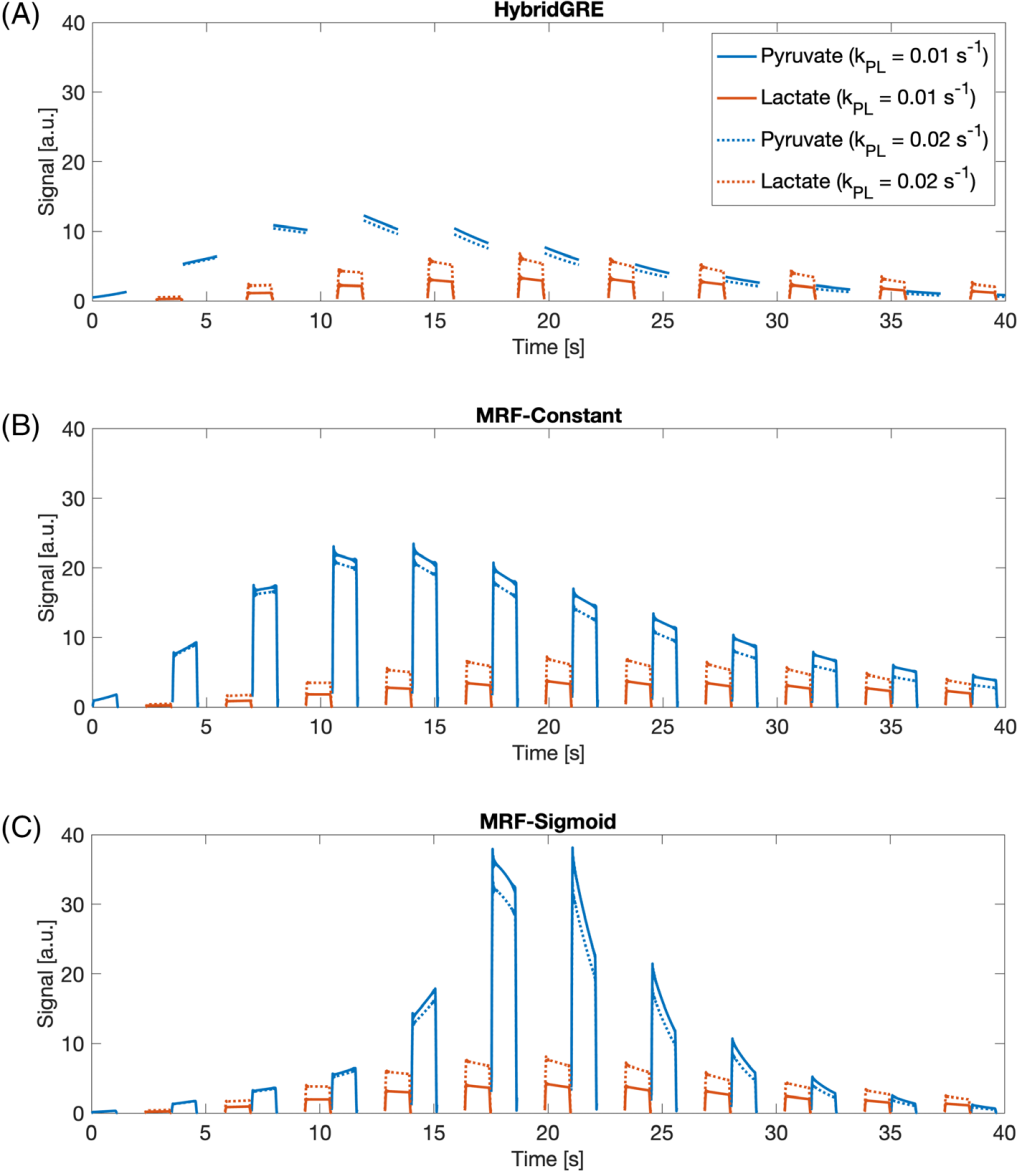
Example simulated signals from each experiment: (A) HybridGRE, (B) MRF‐Constant, and (C) MRF‐Sigmoid. Pyruvate and lactate signal curves at two sample k_PL_ values (0.01 s^−1^, 0.02 s^−1^) illustrate increased pyruvate signal with MRF bSSFP–based methods (b‐c) and comparable lactate signal. Relative changes to signal curves based on changing k_PL_ also vary between GRE‐ and bSSFP‐based experiments.

An MRF dictionary, a set of all fingerprints used for parameter estimation purposes, was created for each described pulse sequence experiment. Each dictionary was populated with combined pyruvate and lactate dynamic signal evolutions across a range of potentially in vivo relevant pyruvate‐to‐lactate (k_PL_) conversion rates (k_PL,min_ = 0.0 s^−1^, k_PL,max_ = 0.1 s^−1^, k_PL,res_ = 0.0001 s^−1^), with each dictionary entry corresponding to a single k_PL_ value (Figure 2). Remainder of input parameters were fixed, with ω_Pyruvate_ = 0 Hz, ω_Lactate_ = 392 Hz, T_1,Pyruvate_ = 30 s, T_1,Lactate_ = 25 s, T_2,Pyruvate_ = 0.5 s, T_2,Lactate_ = 1 s.^6^, ^19^, ^20^, ^21^, ^22^ Pyruvate bolus input timing characteristics were defined reflecting average time for system calibrations following bolus tracking (T_Bolus_Arrival_ = −4 s, T_Bolus_Duration_ = 12 s). Intravoxel field inhomogeneity was defined as LW = 5 Hz.

Parameter estimation for k_PL_ was performed via standard dictionary‐based template‐matching method. Briefly, complex inner products were computed between a given observed signal time course evolution and each normalized dictionary entry. The largest magnitude inner product, from which the associated k_PL_ was derived, was considered to be the best match.

### 2.3 Simulation experiments

The sensitivity of the outlined experimental methods to random noise was assessed using a Monte Carlo evaluation framework with dictionary‐based template matching. Here, for each entry of the prebuilt dictionary, bootstrap added random complex noise, parameterized by σ (SD of both real and imaginary channel noise scaled relative to *M*_Z,Pyruvate_(*t* = 0) or the relaxation‐free total bolus signal) for the set number of iterations (*n* = 10,000), and was subsequently dictionary matched for k_PL_. This process was repeated at three different noise levels, σ = 0.1, 0.2, and 0.3. Example signal curves for all methods and a subset of kinetic rates and noise levels are presented in Figure S1. Relative performances of each experiment were compared on subsets of Monte Carlo results at specific combinations of noise and nominal k_PL_ value via coefficient of variation (CV = (SD / mean) × 100). RF flip angle excitation design for MRF‐Sigmoid and MRF‐Constant were narrowed from a wider range of values and variable excitation schemes evaluated with preliminary (*n* = 100) Monte Carlo experiments at a single value of noise (σ = 0.2).

To characterize the sensitivity of the simulated experiments to fixed fitting parameters, individual sensitivity analyses were performed. The sensitivity of each test parameter was investigated by simulating a subset of metabolite dynamics for a relevant range of test values (*n* = 21) and a nominal k_PL_ value. Simulated test data was matched to a full dictionary of k_PL_ values simulated with the nominal value of the test parameter. Earlier dictionaries, designed for testing fit sensitivity to noise, were utilized as the nominal fit dictionary for each test parameter. The parameters tested for sensitivity were the relaxation times of pyruvate and lactate (T_1_, T_2_), B_1_ scale, B_0_ error, and the B_0_ distribution described as LW‐FWHM. The test simulation parameter minimums, maximums, and resolutions were designed individually for each test parameter and test ranges were centered on nominal values. Pyruvate T_1_ test range was defined by the minimum value T_1,min_ = 20 s, the maximum value T_1,max_ = 40 s, and the test set resolution T_1,res_ = 1 s. Lactate T_1_ test range was defined by the minimum value T_1,min_ = 15 s, the maximum value T_1,max_ = 30 s, and the test set resolution T_1,res_ = 1 s. Pyruvate T_2_ test range was defined by the minimum value T_2,min_ = 0.3 s, the maximum value T_2,max_ = 0.7 s, and the test set resolution T_2,res_ = 0.04 s. Lactate T_2_ test range was defined by the minimum value T_2,min_ = 0.6 s, the maximum value T_2,max_ = 1.4 s, and the test set resolution T_2,res_ = 0.4 s. The test set for B_1_ scale was defined by the minimum value B_1,min_ = 80%, the maximum value B_1,max_ = 120%, and the resolution B_1,res_ = 10%. The B_0_ error or offset test set was defined by the minimum offset B_0_, min = −15 Hz, the maximum offset B_0,max_ = 15 Hz, and the step size B_0,res_ = 1.5 Hz. The test set for the B_0_ distribution LW was defined by the minimum LW_min_ = 0.2 Hz, the maximum test value LW_max_ = 9.8 Hz, and the step size LW_res_ = 0.48 Hz.

### 2.4 Animal studies

Imaging was performed on a 3 T clinical scanner (GE Healthcare MR750, Waukesha, WI) with a dual‐tuned ^1^H/^13^C single‐channel birdcage coil. Studies were conducted on healthy, adult Sprague–Dawley rats according to the University of California, San Francisco Institutional Animal Care and Use Committee–approved protocols. Preparations of the HP solution and the animal were consistent with previous studies,^2^ and polarization was performed using a GE SPINlab 5 T hyperpolarizer. Animals were anesthetized using isoflurane gas (1.5%–2%) and placed supine in the coil on a heated water pad. A total of three identical injections of HP 80 mM [1‐^13^C]pyruvic acid, ˜2.5 mL per injection, were administered to the animal, via a tail vein catheter, and a minimum of 15 min was allowed to elapse between injections. For *n* = 4 studies, each of the three different experiments, described in Table 1, were performed. For a single study (*n* = 1), only two shots were successful, with only two different experiments obtained: MRF‐Sigmoid and HybridGRE.

### 2.5 Analysis

In vivo experimental data (interleaved pyruvate and lactate images) were fit for k_PL_ using the same dictionary‐based template‐matching framework and dictionaries utilized earlier in Monte Carlo evaluations. Kinetic rates were estimated on a per voxel basis within studies and fitting results were plotted against each other to assess for agreement. Pearson correlations, *r*, were calculated for all voxel‐wise paired comparisons as well as paired mean, kidney k_PL_ values per animal. Correlation scores are reported noting the degrees of freedom (n − 2) and significance (*p*). Agreement was further assessed with voxel‐wise Bland–Altman analysis, with mean and 95% confidence interval (CI) metrics.

Anatomical‐based masks, referred to as kidney masks, were manually segmented from reference T_2_‐weighted proton images, yielding a single, multi‐slice binary mask per study. SNR‐based masks were derived on a per experiment basis and defined by pyruvate SNR. The intersection of the proton‐based kidney mask and the pyruvate‐based SNR mask is referred to as the *kidney–SNR mask* and was derived for each experiment of each study individually.

Area‐under‐the‐curve (AUC) images, SNR, and lactate‐to‐pyruvate AUC ratio images were calculated following prior methods.^2^ Dynamic AUC SNR pyruvate images were calculated by cumulatively summing the complex data in the time dimension. The magnitude of the dynamic AUC data was then divided by the SD of the real part of the noise. To compare dynamic AUC SNR of MRF‐Sigmoid or MRF‐Constant to HybridGRE, the experiments were aligned in time by matching the mean lactate time‐to‐peak (TTP) in the kidney–SNR masked images. Lactate TTP values for each experiment were calculated according to previously published methods.^23^ The dynamic AUC SNR images of the MRF experiment were then divided by the TTP matched dynamic AUC SNR HybridGRE images, giving dynamic AUC SNR ratio images. The final reported time series are the mean of the pyruvate dynamic AUC SNR ratio images in the kidney–SNR masked region.

## 3 RESULTS

### 3.1 Simulations

**FIGURE 3:**
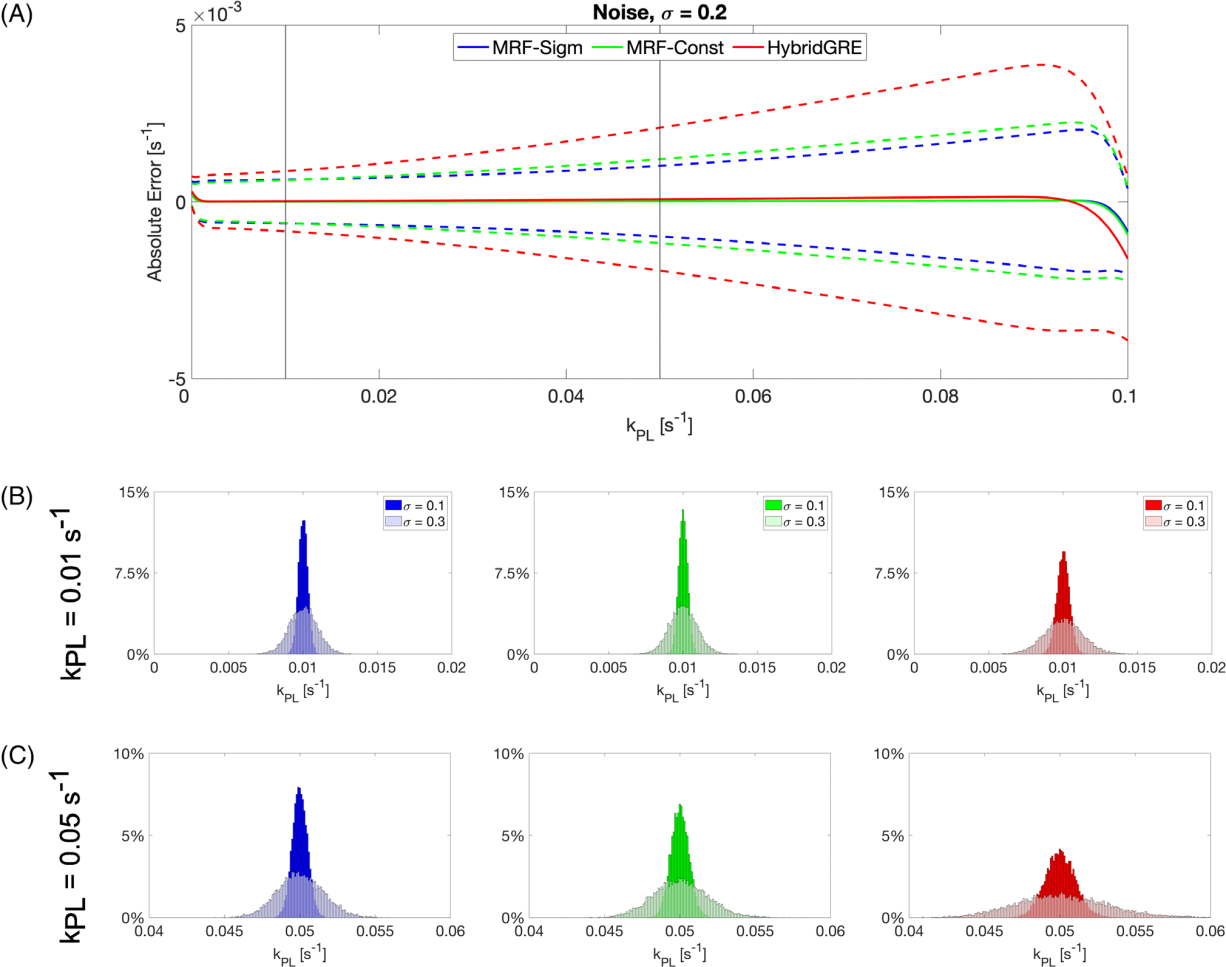
Monte Carlo simulation framework of 10,000 runs was used to evaluate the relative performance of each experimental design: MRF‐Sigmoid, MRF‐Constant, and HybridGRE. Simulated noise was added with SD, σ, relative to $M_{Z,\text{Pyruvate}}\left(t=0\right)$, to match expected levels encountered in vivo. (A) All methods exhibited minimal bias in measurement of k_PL_. MRF‐Sigmoid and MRF‐Constant methods exhibited higher precision across the full dictionary range of k_PL_ in comparison to HybridGRE. (B) Detailed distribution of Monte Carlo results at k_PL_ = 0.01, 0.05 s^−1^ (gray vertical lines within (a)), and noise level σ = 0.1, 0.3 further demonstrate the improvement in measurement precision of bSSFP acquisition–based methods.

Monte Carlo evaluation of all three experiments—MRF‐Sigmoid, MRF‐Constant, and HybridGRE (Figure 3)—exhibited low bias across the majority of k_PL_ values investigated and examined levels of random noise. For MRF‐Sigmoid, MRF‐Constant, and HybridGRE, respectively, 68%, 66%, and 60% of all runs at all simulated k_PL_ values and all noise levels showed bias <1%. Bias of the mean fit for k_PL_ across noise levels (*n* = 3) and all simulation experiments (*n* = 10 000) was less than 0.1% for 86%, 90%, and 37% of results for MRF‐Sigmoid, MRF‐Constant, and HybridGRE, respectively. Fitting values near the upper and lower limits of the dictionary k_PL_ range (0.0 and 0.1 s^−1^) do show bias in mean fit. Both MRF‐Sigmoid and MRF‐Constant methods exhibited lower SDs in absolute fitting error compared to the prior HybridGRE. For instance, within the range of expected normal physiologic values spanning 0.005 to 0.04 s^−1^, the MRF‐Sigmoid method had on average 6% lower SD than the MRF‐Constant method. Relative performance across methods was in line with preliminary Monte Carlo assessment of a range of excitation designs (Figure S1).

Detailed noise sensitivity analysis (Figure 3B,C) demonstrates relatively normal distributed fitting results with minimal skew centered on the nominal value in both low and high noise scenarios (bias <1%). At k_PL_ = 0.01 s^−1^, MRF‐Sigmoid, MRF‐Constant, and HybridGRE bias values were 0.06%, 0.04%, and 0.08%, respectively, at σ = 0.1; and 0.18%, 0.14%, and 0.35% at σ = 0.3. For simulation experiments with true k_PL_ = 0.05 s^−1^, MRF‐Sigmoid, MRF‐Constant, and HybridGRE bias values were 0.02%, 0.01%, and 0.05%, respectively, at σ = 0.1; and 0.07%, 0.08%, and 0.31% at σ = 0.3. At both levels of added noise and k_PL_ values, MRF‐Sigmoid and MRF‐Constant experiments show lower variance compared to HybridGRE. For MRF‐Sigmoid, MRF‐Constant, and HybridGRE, respectively, at k_PL_ = 0.01 s^−1^, CVs were 3.09%, 3.04%, and 4.25% at σ = 0.1; and 9.24%, 9.08%, and 12.7% at σ = 0.3. For MRF‐Sigmoid, MRF‐Constant, and HybridGRE, respectively, at k_PL_ = 0.05 s^−1^, CVs were 1.00%, 1.19%, and 2.01% at σ = 0.1; and 3.00%, 3.56%, and 6.07% at σ = 0.3. A subset of noisy (σ = 0.2) iterations (*n* = 100) were also fit with direct curve fitting (MatLab lsqnonlin function, MathWorks) to compare current standard kinetic rate modeling techniques directly to dictionary‐based template‐matching approach. Maximum bias of direct curve fitting results of MRF‐Sigmoid was 4.3%, whereas the maximum bias of the dictionary‐based estimations was 1% (Figure S3).

**FIGURE 4:**
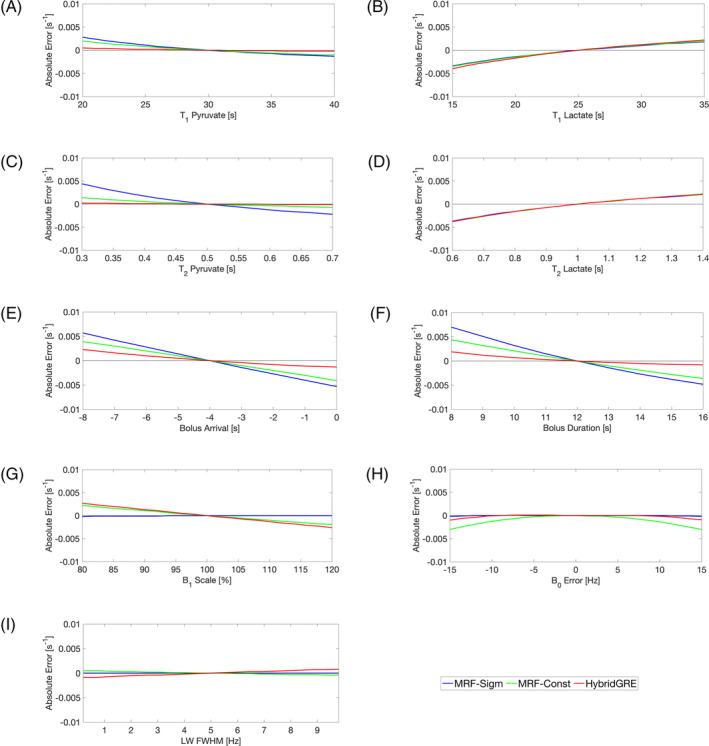
Parameter sensitivity analysis of k_PL_ estimation in the setting of dictionary non‐matched parameter errors. Simulated experimental signals with k_PL_ = 0.02 s^−1^ but variation across nonestimated parameters are matched against preconstructed dictionary varied across k_PL_ but otherwise fixed nonestimated parameters. The absolute error between simulated and fit k_PL_ is reported for (A) pyruvate T_1_, (B) lactate T_1_, (C) pyruvate T_2_, (D) lactate T_2_, (E) bolus arrival relative to acquisition start, (F) bolus duration, (G) B_1_ relative scale, (H) B_0_ error, and (I) LW‐FWHM. The simulated signals including sensitivity offsets were fit with the dictionary defined by the nominal sensitivity value for each experimental design independently for MRF‐Sigmoid, MRF‐Constant, and HybridGRE, respectively.

Fitting parameter sensitivities illustrating the expected effects on kinetic rate estimation results in the setting of dictionary simulation parameter deviation from the actual experiment conditions are shown in Figure 4. Specifically, underestimation of T_1,Pyruvate_ resulted in overestimation of k_PL_ in both MRF‐Sigmoid (max error = 0.003 s^−1^) and MRF‐Constant methods (max error = 0.002 s^−1^), whereas HybridGRE method (max error = 0.0005 s^−1^) is less sensitive to the variation of this parameter. In contrast, underestimation of T_1,Lactate_ resulted in similar degrees of underestimation of k_PL_ with all three methods (MRF‐Sigmoid: max error = −0.003 s^−1^, MRF‐Constant: max error = −0.004 s^−1^, HybridGRE: max error = −0.004 s^−1^). Underestimation of T_2,Pyruvate_ impacted HybridGRE fit results the least (max error = 0.0001 s^−1^), whereas it resulted in overestimation of k_PL_ with MRF‐Constant (max error = 0.001 s^−1^) and MRF‐Sigmoid methods (max error = 0.004 s^−1^). Underestimation of T_2,Lactate_ had similar underestimation of k_PL_ between all three methods (max error = −0.004 s^−1^). MRF‐Sigmoid method displayed the highest sensitivity to bolus arrival time and bolus duration, in cases of both under‐ and overestimation (bolus arrival max error = 0.006 s^−1^, bolus duration max error = 0.007 s^−1^). MRF‐Constant showed less sensitivity to the two bolus parameters with max error across both analyses of 0.004 s^−1^. The HybridGRE method was the least sensitive to bolus arrival and bolus duration with max error of 0.002 s^−1^. Across sensitivity to B_1_ scale, B_0_ field errors, and field inhomogeneity (LW), MRF‐Sigmoid had the lowest maximum error of −0.0002 s^−1^, −0.0002 s^−1^, and 0.0 s^−1^, respectively. The MRF‐Constant method had maximum errors of 0.002 s^−1^, −0.003 s^−1^, and 0.001 s^−1^, respectively, for results of B_1_ scale, B_0_ error, and LW. HybridGRE sensitivity results were comparable to MRF‐Constant, with max errors of 0.003 s^−1^, −0.001 s^−1^, and ‐0.001 s^−1^. When considered as relative error, most sensitivity parameters investigated exhibited less than 25% error, excluding only bolus arrival time and bolus duration (see Figure S4).

### 3.2 Animal studies

**FIGURE 5:**
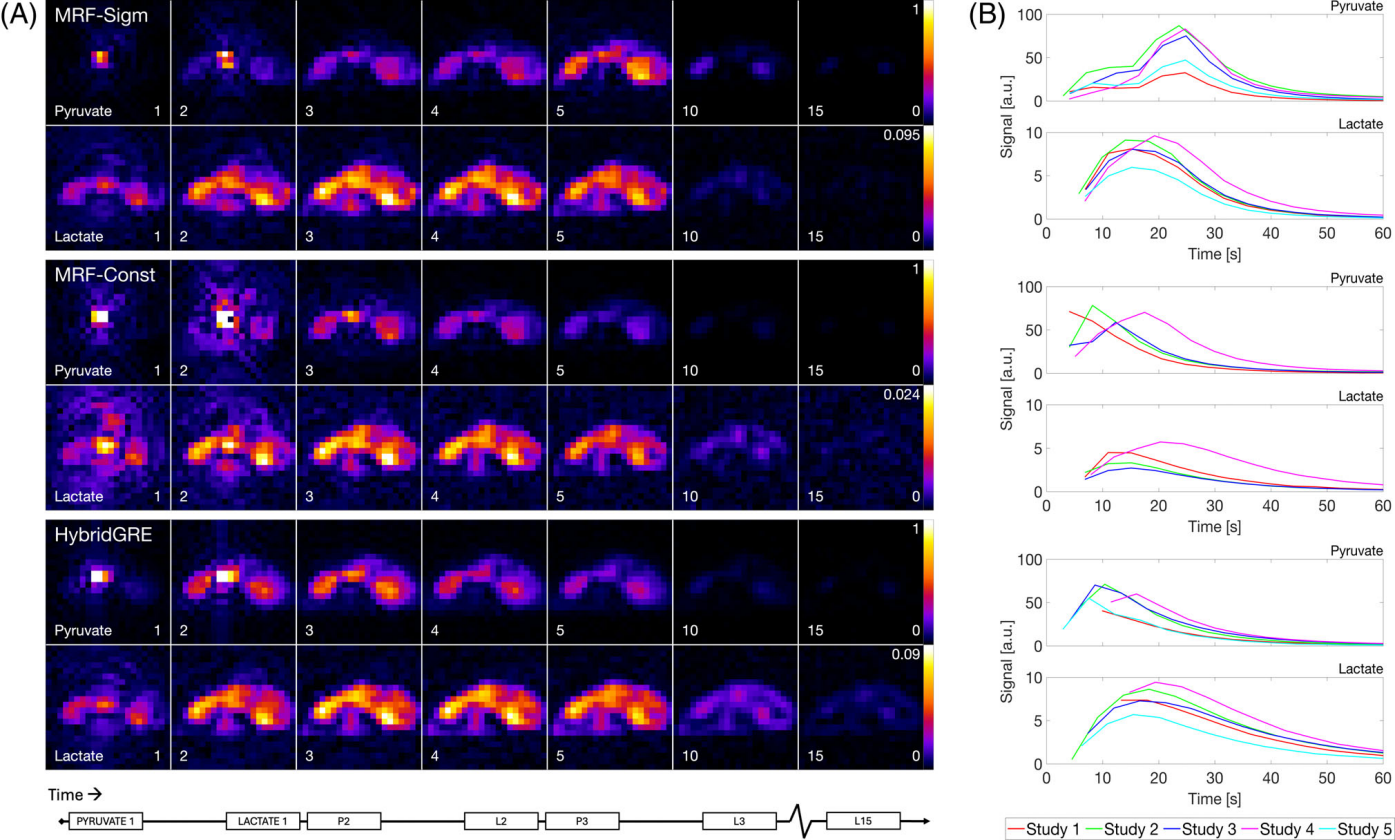
Example dynamic ^13^C images acquired in vivo on rat kidney using MRF‐Sigmoid, MRF‐Constant, and HybridGRE. (A) Relative differences in the dynamic images between methods are shown for the center slice (z = 9). Lactate images are comparable across all methods, whereas pyruvate dynamic images are distinct, especially for the MRF‐Sigmoid method because of the sigmoid excitation design. (B) The mean signal dynamics of SNR‐based masked images across all studies are plotted for the first 60s, postinjection start, and further illustrate the differences in dynamics and relative signal. ^13^C, Carbon‐13.

Small animal studies were performed to compare the examined methods and the proposed bSSFP‐type MRF acquisition to prior HybridGRE. Volumetric imaging allowed intra‐animal comparisons of pyruvate and lactate perfusion and spatial distribution of metabolites. Dynamic images and mean signal curves (Figure 5A,B) illustrate the signal evolution observed in vivo of all three methods. MRF‐Sigmoid with sigmoid‐shaped RF excitation envelope shows delayed pyruvate time‐to‐peak signal compared to other methods, consistent with simulation. Lactate images and mean signal curves in the MRF‐Sigmoid method also display higher peak signal values relative to correlate pyruvate data when compared to the MRF‐Constant, which are on par with the prior HybridGRE method.

**FIGURE 6:**
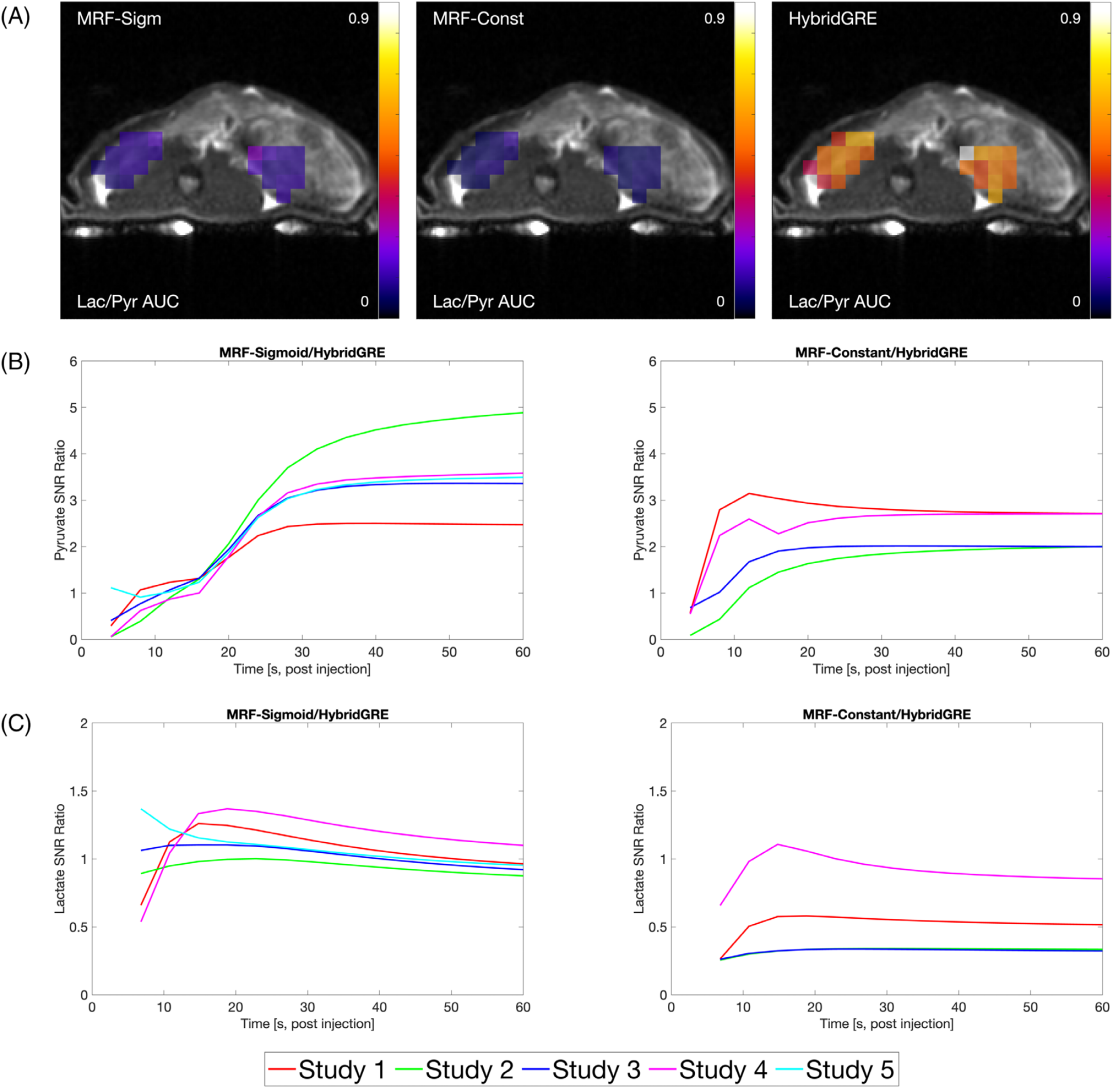
Example lactate/pyruvate AUC maps in vivo (A) with lower lactate/pyruvate ratio observed in MRF‐Sigmoid and MRF‐Constant methods compared to HybridGRE, predominantly due to higher preserved pyruvate SNR related to bSSFP‐based acquisition. This is further demonstrated by (B) cumulative pyruvate signal from kidney voxels with resulting 3.5‐fold for MRF‐Sigmoid and 2.4‐fold for MRF‐Constant relative increased SNR as compared to HybridGRE. Cumulative lactate signal ratios (C) indicate that MRF‐Sigmoid and HybridGRE have similar lactate SNR, whereas on average SNR of lactate in MRF‐Constant experiments is lower than HybridGRE. AUC, area under the curve.

The relative signal amplitudes and SNR of the methods are compared in Figure 6. HybridGRE method, which used high signal efficiency SSFP‐type acquisition of lactate, with lower signal efficiency GRE‐type acquisition for pyruvate, demonstrate higher absolute lactate‐to‐pyruvate AUC ratio compared to the MRF methods, which used only bSSFP‐type acquisition (Figure 6A). Distribution and value of lactate‐to‐pyruvate AUC ratios for MRF‐Sigmoid and MRF‐Constant methods are consistent. Mean signal within kidney voxels showed higher SNR of acquired pyruvate images when acquired with MRF‐Sigmoid and MRF‐Constant, compared to HybridGRE (Figure 6B). The highest relative increase in dynamic pyruvate SNR was seen with MRF‐Sigmoid method, which had an average 3.1‐fold gain at 30 s of acquisition and an average 3.5‐fold SNR gain at 60 s across all studies compared to HybridGRE. MRF‐Constant method also showed gains in dynamic pyruvate SNR with a 2.3‐fold cumulative increase at 30 s of acquisition and 2.4‐fold gain at 60 s of acquisition compared to HybridGRE. Comparisons of lactate SNR in kidney voxels across the acquisition window (Figure 6C) indicated that MRF‐Constant lactate images had lower SNR relative to HybridGRE on average, with an SNR ratio of 0.54 at 30 s and 0.50 at 60 s of acquisition. MRF‐Sigmoid lactate image SNR more closely matched that of HybridGRE acquisitions, with an SNR ratio of 1.10 at 30 s and 0.94 at 60 s compared to HybridGRE.

**FIGURE 7:**
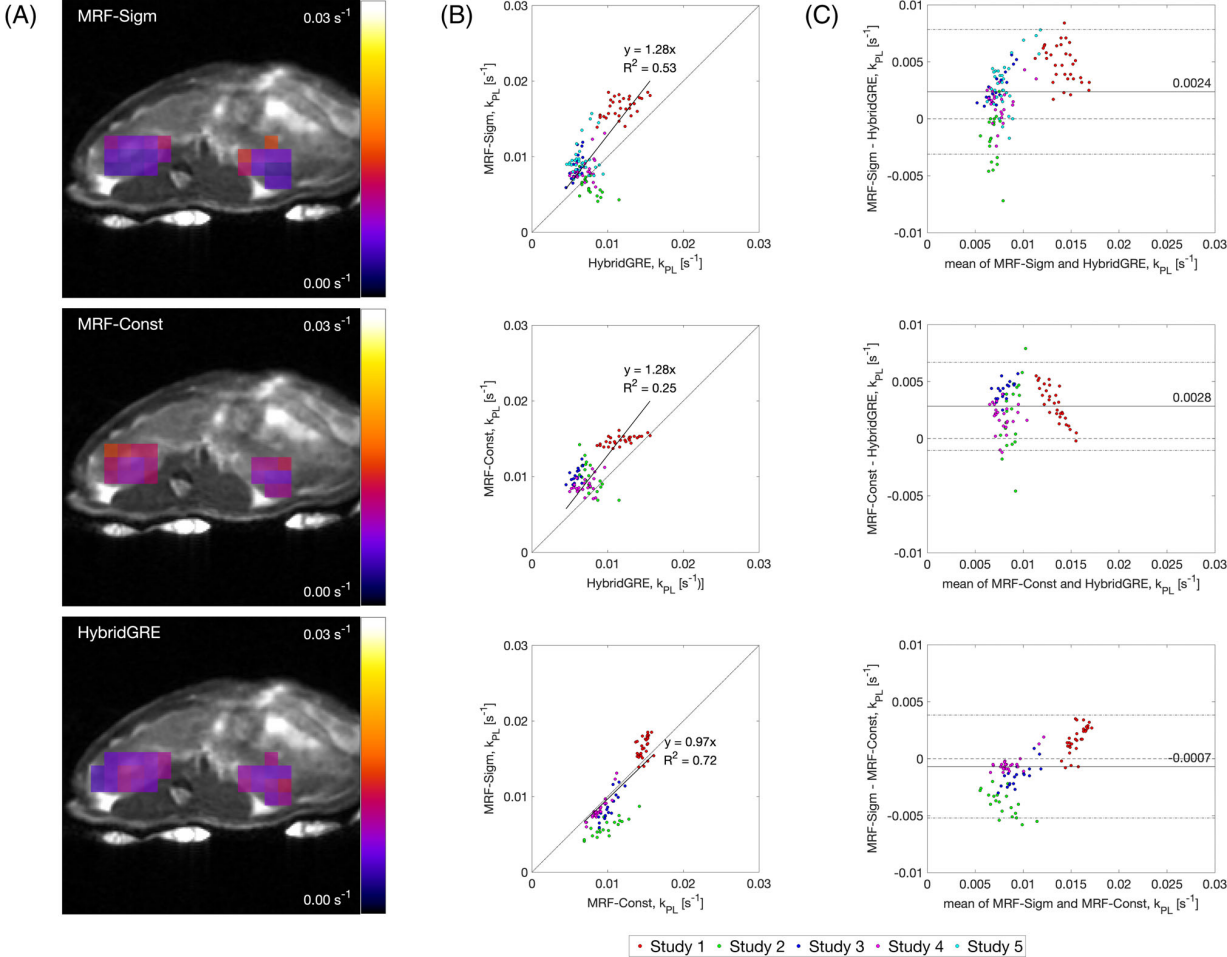
Voxel‐wise k_PL_ rate estimation between experimental methods. (A) Example k_PL_ parameter maps for the center slice (z = 9) of study 3. (B) Voxel‐wise comparison between MRF‐Sigmoid and HybridGRE methods show positive linear correlation (best fit slope = 1.28, *R* ^2^ = 0.53), and the Bland–Altman analysis between the two methods show a positive average discrepancy of 0.0024 s^−1^ from MRF‐Sigmoid to HybridGRE across all studies (*n* = 5). Voxel‐wise comparison between MRF‐Constant and HybridGRE methods also show a positive linear correlation (best fit slope = 1.28, *R* ^2^ = 0.25) and Bland–Altman analysis results in a positive average discrepancy of 0.0028 s^−1^ from MRF‐Constant to HybridGRE across all applicable studies (*n* = 4). Voxel‐wise comparison between MRF‐Sigmoid and MRF‐Constant methods show a positive linear correlation (best fit slope = 0.97, *R* ^2^ = 0.72), and Bland–Altman analysis results in a negative average discrepancy of 0.0007 s^−1^ across all applicable studies (n = 4)

Comparisons of kinetic rate fitting are shown in Figure 7. Example k_PL_ kinetic rate maps from the same animal are shown in Figure 7A. The maps demonstrate similarities in distributions and values in the kidneys across methods. Voxel‐wise comparison (Figure 7B) with Bland–Altman analysis (Figure 7C) of k_PL_ from kidney mask show positive, linear correlation of kinetic rate measurements between methods, with *R* ^2^ = 0.53 between MRF‐Sigmoid and HybridGRE, *R* ^2^ = 0.25 between MRF‐Constant and HybridGRE, and *R* ^2^ = 0.72 between MRF‐Sigmoid and MRF‐Constant. Pearson correlation coefficients were also calculated for each voxel‐wise comparison and on a per animal basis using the mean kidney k_PL_ value. MRF‐Sigmoid and HybridGRE were strongly positively correlated (*r* [131] = 0.73, *p* < 0.0001). Similarly, MRF‐Constant and HybridGRE were found to be highly positively correlated (*r* [98] = 0.74, *p* < 0.0001), and MRF‐Sigmoid and MRF‐Constant were also significantly positively correlated (*r* [98] = 0.91, *p* < 0.0001). Average per‐animal correlations were strong. The correlation between MRF‐Sigmoid and HybridGRE was *r* (3) = 0.74, *p* < 0.01; between MRF‐Constant and HybridGRE the correlation was *r* (2) = 0.91, *p* < 0.01; and lastly, the correlation between MRF‐Sigmoid and MRF‐Constant was *r* (2) = 0.93, *p* < 0.01. Additional Bland–Altman comparison is provided in Figure 7C, which shows mean bias between MRF‐Sigmoid and HybridGRE methods was 0.0024 s^−1^, CI = (−0.0031, 0.0078). Average bias between MRF‐Constant and HybridGRE was 0.0028 s^−1^, CI = (−0.0010, 0.0067). Finally, average bias between MRF‐Sigmoid and MRF‐Constant was 0.0007 s^−1^, CI = (−0.0052, 0.0038).

## 4 DISCUSSION

This work demonstrates the novel application of the MRF framework to HP ^13^C acquisition methods and pulse sequence design. Within this framework, bSSFP‐type pyruvate and lactate acquisition with customized flip angle approach MRF methods are compared against previous MS‐3DSSFP method due to the high relative SNR efficiency of bSSFP‐type acquisitions. Numerical simulations were used to examine the accuracy of these methods under a variety of noise and expected physiologic conditions. The sensitivity of these methods to numerous potential error sources was assessed. The feasibility and accuracy of these methods are shown in rat kidneys at 3 T.

The MRF framework served several key purposes in this work. First, it serves as a consistent and robust parameter estimation method independent of pulse sequence design. Numerous kinetic rate estimation methods have been proposed for ^13^C methods over the years, including direct curve fitting to kinetic exchange models.^5^, ^24^, ^25^, ^26^, ^27^ However, these methods are computationally intensive and time‐consuming, and their performance can depend on constraint and initialization choices. Other approaches, such as ratiometric modeling,^28^ lactate/pyruvate AUC ratio, and time‐to‐peak measurements, have been examined over the years^6^, ^23^ but depend on pulse sequence design, parameter choices, and physiologic conditions. MRF, using a precomputed dictionary with direct template‐matching approach, is unique in that after initial precomputation of the dictionary, template matching is fast and efficient. Under our framework, generating a 100‐entry MRF‐Sigmoid or MRF‐Constant dictionary ran in under 15 min, and generating a comparable sized HybridGRE dictionary ran in under 10 min. Previous analysis using MRF in MRS has shown that under noise‐free conditions, parameter estimation using a template‐matching dictionary approach generates errors that are approximately the dictionary step size.^12^ In the current work, we further show that under random complex noise conditions, template‐matching estimation results in approximately normally distributed nonbiased parameter estimation, with deviations only near the bounds of the used dictionary. Template‐matching parameter estimation with MRF‐Sigmoid was compared to standard direct curve fitting and showed lower worst‐case bias (dictionary fit, maximum bias = 1%, direct fit, maximum bias = 4.3%) when fitting noisy (σ = 0.2) data; this minimal difference between fitting methods was also confirmed for in vivo data (Figure S3). Monte Carlo experiments were performed on a standard workstation, with *n* = 10 000 trials completed, on average, in approximately 220 ms. Compared to our naïve implementation of direct curve fitting (average 20 min per fit computation), template matching quickly outpaces the number of computations possible in a given time frame, even when including the computation time of a large 1000‐entry dictionary (Figure S3). This speed allows for rapid analysis of performance differences, such as the robustness to noise improvement of MRF‐Sigmoid over MRF‐Constant, as well as enabling fast reconstruction of experimental data. Additionally, the MRF framework is flexible regarding choice of underlying model and model parameters. Whereas this work used the solution of the Bloch‐McConnell equations for a one‐way, two‐pool kinetic exchange model with gamma input function, the underlying choices, for instance of different compartment models, input functions, or metabolites (i.e., bicarbonate, alanine), can be readily adapted. Other potential confounding effects such as flow or perfusion could also be incorporated into the signal model. Use of dictionary‐based template matching to fit experimental data to the modeled signal differs from prior work of this group that employed nonlinear least squares fitting for voxel‐wise and regional quantification of kinetic rates, often with the use of *input‐less* style estimation.^5^, ^6^ Input‐less fitting is particularly robust to T_Bolus Arrival_ and T_Bolus Duration_, whereas the current MRF framework is more akin to fitting with fixed bolus characteristics and therefore sensitive to bolus timing parameters. In the future, simultaneous estimation of kinetic rate and bolus timing may be possible with the MRF framework.

In multiparametric quantitative proton MRF, bSSFP‐type acquisitions have historically been attractive due to their high SNR efficiency. Initially, this was used for rapid simultaneous T_1_, T_2_, and proton density quantification, which has since been expanded to a vast array of applications.^29^ In spectroscopy, bSSFP SNR efficiency has been demonstrated in Phosphorus‐31 applications. Previously, our group has demonstrated adoption of bSSFP‐type acquisition for lactate‐selective imaging while maintaining GRE‐type acquisitions for pyruvate‐selective imaging, resulting in ˜2.5‐fold increased SNR in lactate. In the current work, we show the potential benefit of bSSFP‐type acquisition in pyruvate acquisition, resulting in increased pyruvate SNR of 3.5‐fold and 2.4‐fold for MRF‐Sigmoid and MRF‐Constant, respectively. Future studies will need to be performed to confirm if these improvements persist in pathology.

Adoption of bSSFP‐type acquisition schemes introduces possible sensitivity to several potential error sources. Due to its well‐established T_2_/T_1_ sensitivity, bSSFP‐type acquisition signal evolutions for a given metabolite will unavoidably be affected by their intrinsic T_1_ and T_2_ relaxation rates.^5^ In this work, T_1_ and T_2_ are fixed in the signal model, and the impact of this approach is shown through simulation. Errors in model assumptions in each of T_1,Lactate_, T_2,Lactate_, and T_1,Pyruvate_ show mild impact on k_PL_ quantification, where even 60% overestimation of these properties results in less than ˜20% quantification error. T_2,Pyruvate_ error sensitivity was slightly worse, particularly for MRF‐Sigmoid, with up to 22% error in k_PL_ estimation given 60% overestimation in T_2,Pyruvate_. One potential option of mitigating this sensitivity is through simultaneous quantification of T_1_ and T_2_, which could be theoretically built into the parameter estimation dictionary as additional dimensions. In fact, previously, the inherent T_1_ and T_2_ sensitivity of bSSFP‐type acquisition has been exploited to directly quantify T_1_ and T_2_ of single metabolites under the assumption of no kinetic exchange.^20^ However, robust simultaneous quantification of kinetic and relaxation rate parameters is challenging and difficult to validate.

With the flexibility of the MRF framework, there is a near infinite design space of potential pulse sequence choices. Initial proton MRF sequences featured pseudorandom flip angle and TR designs,^11^ with a vast variety of developed methods since. More recently, smoothly ramped flip angles have been used to drive transient magnetization smoothly and efficiently. Sequence optimization remains an open debate, but studies suggest ordered, structured designs outperform random/pseudorandom designs.^30^, ^31^ Flip angle design for the current SNR‐limited application, similarly, was constrained to smooth flip angle patterns.

Variable flip angle approaches, with low initial flip angles to preserve signal, have been explored previously with GRE‐type approaches^6^, ^8^, ^32^ and remain attractive, with a logical basis of preserving HP pyruvate magnetization prior to underlying kinetic rate exchange and employing high flip angles later to effectively sample signal before magnetization decay. In this work, preliminary Monte Carlo simulations suggested high relative performance of the proposed sigmoid excitation pattern among a group of candidate sequence designs (Figure S2). Accordingly, in vivo experiments show a 350% increase in pyruvate SNR, whereas lactate SNR slightly decreases (6% decrease). By comparison, the MRF‐Constant method that implements bSSFP‐type acquisition without variable flip angle approach achieved a 240% increase in pyruvate SNR but a 50% decrease in lactate SNR. The implementation of our variable flip angle approach therefore increases SNR of both pyruvate and lactate. Lactate cumulative SNR varied between the different methods despite identical acquisition between all three methods, likely related to differences in loss of HP pyruvate magnetization.

Generalized optimization remains challenging because sequence design requires tradeoffs between performance of difference tissue metabolic rates that are expected to be encountered, and sensitivity to nonestimated parameters. Whereas Monte Carlo simulations showed 25%–50% improved k_PL_ CV with either MRF‐Sigmoid or MRF‐Constant compared to HybridGRE, comparison between MRF‐Sigmoid and MRF‐Constant showed varying k_PL_ estimation robustness. At lower simulated k_PL_ values, MRF‐Constant estimation results showed slightly lower bias and lower CV across all noise values compared to MRF‐Sigmoid. At higher k_PL_, where pathology is favored to be reflected, MRF‐Sigmoid results have approximately 15% lower CV across all noise levels compared to MRF‐Constant results. Bias results also indicate some inconsistency in relative performance of bSSFP‐type experiments. At higher k_PL_ and lower noise, MRF‐Constant exhibits lower bias than MRF‐Sigmoid, whereas at higher noise, MRF‐Sigmoid has lower fit bias. Furthermore, MRF‐Sigmoid, with variable flip angle approach, was the most sensitive to bolus arrival and duration (Figure 4), which has been noted with previous variable flip angle methods.^6^ This can be alleviated with more precise control of the bolus injection as well as through the use of bolus tracking and automated acquisition timing.^33^

For purposes of this study, sequence design choice was limited to constant TR bSSFP due to the desire to limit potential off‐resonance excitation similar to previous methods.^2^, ^3^, ^16^ For each experiment method, pyruvate‐ and lactate‐specific images were acquired with a delay between the two images to imitate the future possibility of imaging a third metabolite. The MRF framework could easily be extended to include other metabolites such as ^13^C‐bicarbonate and [1‐^13^C]alanine or perfusion modeling based on [^13^C, ^15^N_2_]urea.

In the current work, the limits of achievable spatial resolution were not explored, despite the well‐recognized potential of MRF framework methods to make use of spatial undersampling. While MRF methods are often robust to undersampling artifacts, the choice of undersampling strategy still results in the potential for spatially dependent parameter estimation error.^34^ Numerous reconstruction methods have been proposed to this effect, including iterative, low rank, and deep learning approaches. The potential additional confounding effects of undersampling and combination with additional advanced reconstruction techniques will be investigated in future work.

## 5 CONCLUSION

Hyperpolarized [1‐^13^C]pyruvate MRI acquisitions are SNR‐limited, and accurate and precise estimation of kinetic rates can be computationally intensive, complex, and sensitive to noise and underlying parameters. This work demonstrates the feasibility of implementing bSSFP‐type acquisitions for pyruvate and downstream metabolites to increase overall SNR. Additionally, implementation of variable flip angle excitation of pyruvate, inspired by MRF applications to MRS and HP signal optimization, further increase SNR of pyruvate and lactate. The use of the MRF framework for k_PL_ estimation demonstrates the feasibility of dictionary‐based template matching, and Monte Carlo simulations highlight the improvements to both precision and accuracy of MRF‐Sigmoid and MRF‐Constant experiments over the prior HybridGRE experiment. In vivo results further demonstrate voxel‐wise and regional correlations of k_PL_ estimates between methods. Overall, the MRF framework with bSSFP acquisition and variable flip angles can be used to accurately estimate physiologically relevant k_PL_ and improve cumulative SNR. In the future, this work can be expanded to accommodate underlying models of higher complexity, additional metabolites, or spatial undersampling to further improve the quality of HP studies and qualitative metrics.

## CONFLICT OF INTEREST STATEMENT

Peder Larson has funding from GE HealthCare.

## Supporting information


**Figure S1.** Example simulated signal curves from Monte Carlo simulation framework results with two levels of added noise and two values of kPL (0.01 s^−1^, 0.05 s^−1^). (A–C) A subset of signal curves computed with low level noise and low value of kPL (s = 0.1, kPL = 0.01 s^−1^) across all methods: HybridGRE, MRF‐Constant and MRF‐Sigmoid. Sample noise‐added simulations of (d)‐(f) higher noise and low metabolic exchange (s = 0.3, kPL = 0.01 s^−1^), (G–I) low noise and moderate metabolic conversion (s = 0.1, kPL = 0.05 s^−1^), and (J–L) higher noise and moderate metabolic conversion (s = 0.3, kPL = 0.05 s^−1^), also show the relative variation across methods.
**Figure S2.** Exploratory Monte Carlo evaluations (*n* = 1000) of constant and variable flip angle schemes were performed, (A) mean relative error in and (B) standard deviation of kPL estimations are reported. Box plot of Monte Carlo result distributions of various flip angle schemes at values of kPL = 0.01 (C), 0.05 (D) and 0.08 s^−1^ (E), respectively.
**Figure S3.** Kinetic rate estimation results from direct curve fitting using MATLAB lsqnonlin function for (A) MRF‐Sigmoid under noise‐free conditions and (B) under Monte Carlo evaluation (*n* = 100, s = 0.2). (C) Comparison of kinetic rate estimation of noisy data with direct curve fitting and dictionary‐based template matching, showing average bias of −0.0002 s^−1^. Distribution of sequential fitting computation times are reported per single fit for (D) direct curve fitting and (E) dictionary‐based template matching. (f) Expected combined computation time for dictionary‐creation and template matching remains relatively constant with increasing numbers of estimations compared to direct curve fitting (requiring no upfront reference computations) which linearly increases. Voxel‐wise kPL rate estimation for in vivo datasets fit via dictionary‐based template matching and direct curve fitting directly compared via correlation plots for (G) MRF‐Sigmoid, (H) MRF‐Constant, and (I) HybridGRE experiments.
**Figure S4.** Monte Carlo simulation results across the full dictionary reported as error relative to the ground truth kPL value. The relative error between simulated and fit kPL is also reported for (B) pyruvate T_1_, (C) lactate T_1_, (D) pyruvate T_2_, (E) lactate T_2_, (F) bolus arrival relative to acquisition start, (G) bolus duration, (H) B1 relative scale, (I) B0 error and (J) LW full width half max. The simulated signals including sensitivity offsets were fit with the dictionary defined by the nominal sensitivity value for each experimental design independently for MRF‐Sigmoid, MRF‐Constant, and HybridGRE, respectively.
