# Supplementary material for: A flexible MRF approach to improve kinetic rate estimation with bSSFP‐based hyperpolarized [1‐ 13C]pyruvate MRI
Source: Magn Reson Med. 2025 Mar 4;93(6):2263–77. doi: 10.1002/mrm.30466 (PMC11971489; doi:10.1002/mrm.30466)
Supplement: Supplementary file 1 — Figure S1. Example simulated signal curves from Monte Carlo simulation framework results with two levels of added noise and two values of kPL (0.01 s−1, 0.05 s−1). (A–C) A subset of signal curves computed with low level noise and low value of kPL (s = 0.1, kPL = 0.01 s−1) across all methods: HybridGRE, MRF‐Constant and MRF‐Sigmoid. Sample noise‐added simulations of (d)‐(f) higher noise and low metabolic exchange (s = 0.3, kPL = 0.01 s−1), (G–I) low noise and moderate metabolic conversion (s = 0.1, kPL = 0.05 s−1), and (J–L) higher noise and moderate metabolic conversion (s = 0.3, kPL = 0.05 s−1), also show the relative variation across methods. Figure S2. Exploratory Monte Carlo evaluations (n = 1000) of constant and variable flip angle schemes were performed, (A) mean relative error in and (B) standard deviation of kPL estimations are reported. Box plot of Monte Carlo result distributions of various flip angle schemes at values of kPL = 0.01 (C), 0.05 (D) and 0.08 s−1 (E), respectively. Figure S3. Kinetic rate estimation results from direct curve fitting using MATLAB lsqnonlin function for (A) MRF‐Sigmoid under noise‐free conditions and (B) under Monte Carlo evaluation (n = 100, s = 0.2). (C) Comparison of kinetic rate estimation of noisy data with direct curve fitting and dictionary‐based template matching, showing average bias of −0.0002 s−1. Distribution of sequential fitting computation times are reported per single fit for (D) direct curve fitting and (E) dictionary‐based template matching. (f) Expected combined computation time for dictionary‐creation and template matching remains relatively constant with increasing numbers of estimations compared to direct curve fitting (requiring no upfront reference computations) which linearly increases. Voxel‐wise kPL rate estimation for in vivo datasets fit via dictionary‐based template matching and direct curve fitting directly compared via correlation plots for (G) MRF‐Sigmoid, (H) MRF‐Constant, and (I) HybridGRE expe [file MRM-93-2263-s001.docx]

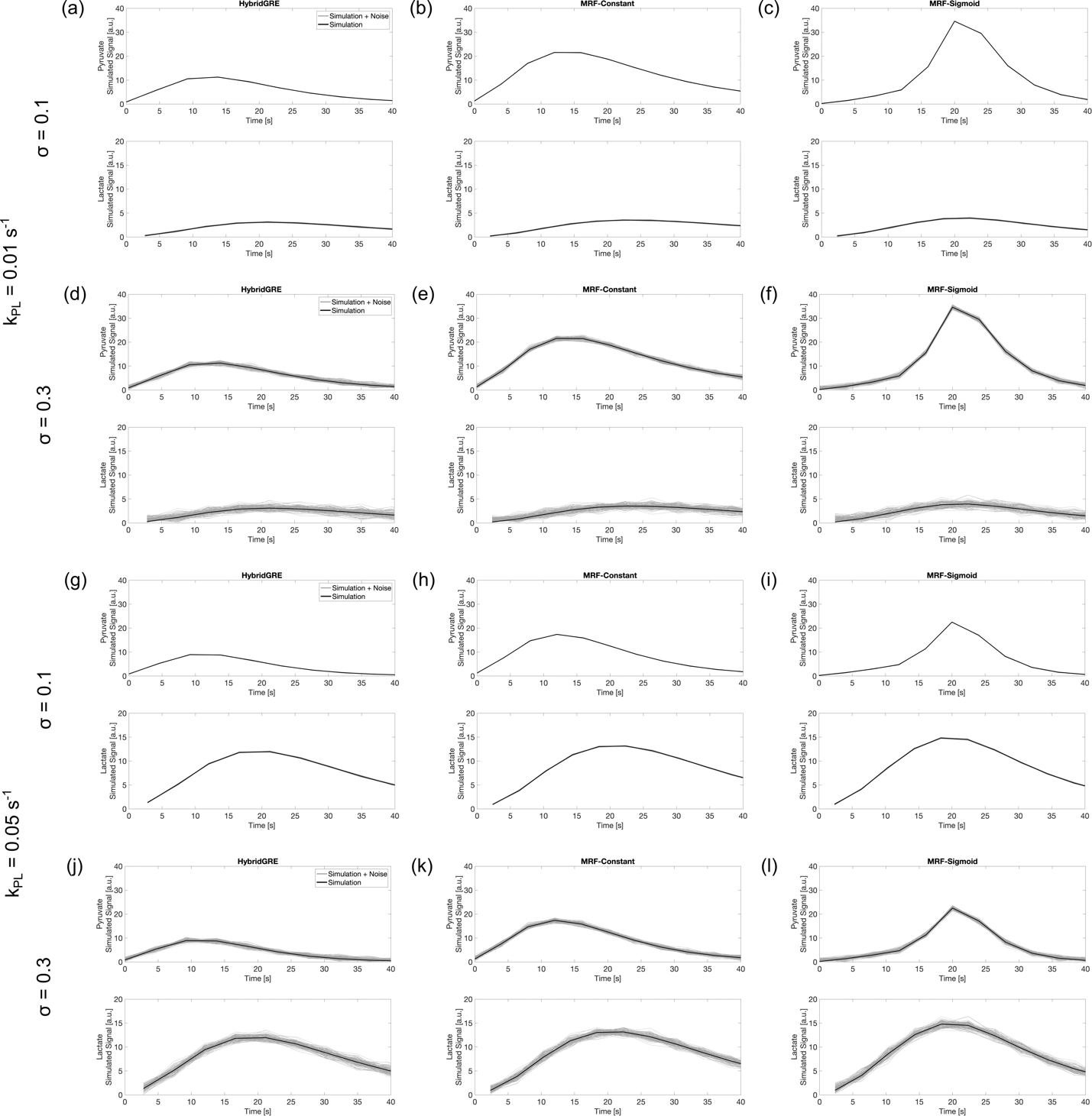


Figure S1: Example simulated signal curves from Monte Carlo simulation framework results with two levels of added noise and two values of kPL (0.01s-1, 0.05s-1). (a)-(c) A subset of signal curves computed with low level noise and low value of kPL (s = 0.1, kPL = 0.01s-1) across all methods: HybridGRE, MRF-Constant and MRF-Sigmoid. Sample noise-added simulations of (d)-(f) higher noise and low metabolic exchange (s = 0.3, kPL = 0.01s-1), (g)-(i) low noise and moderate metabolic conversion (s = 0.1, kPL = 0.05s-1), and (j)-(l) higher noise and moderate metabolic conversion (s = 0.3, kPL = 0.05s-1), also show the relative variation across methods.


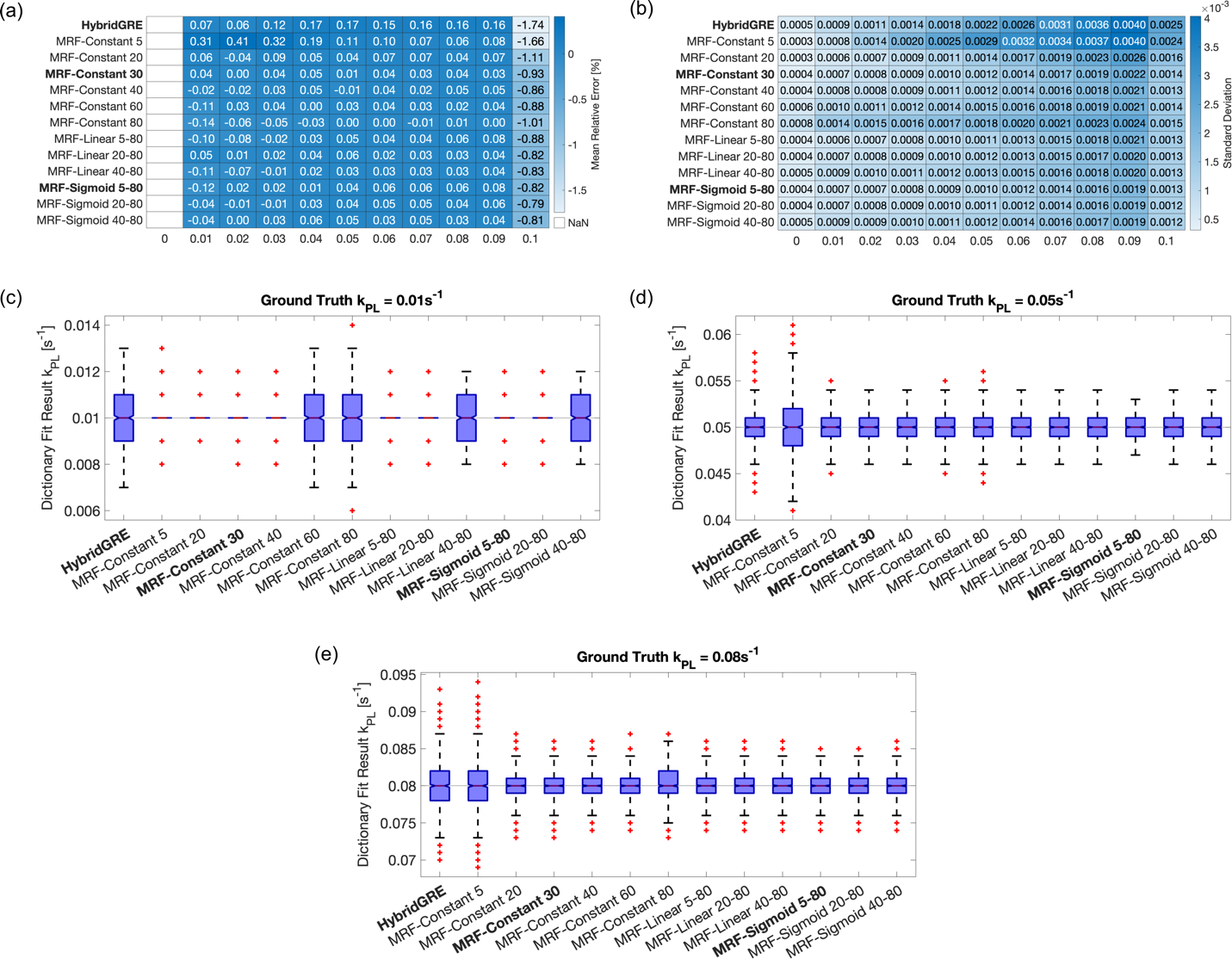


Figure S2: Exploratory Monte Carlo evaluations (n=1000) of constant and variable flip angle schemes were performed, (a) mean relative error in and (b) standard deviation of kPL estimations are reported. Box plot of monte carlo result distributions of various flip angle schemes at values of kPL = 0.01 (c), 0.05 (d) and 0.08s-1 (e), respectively.


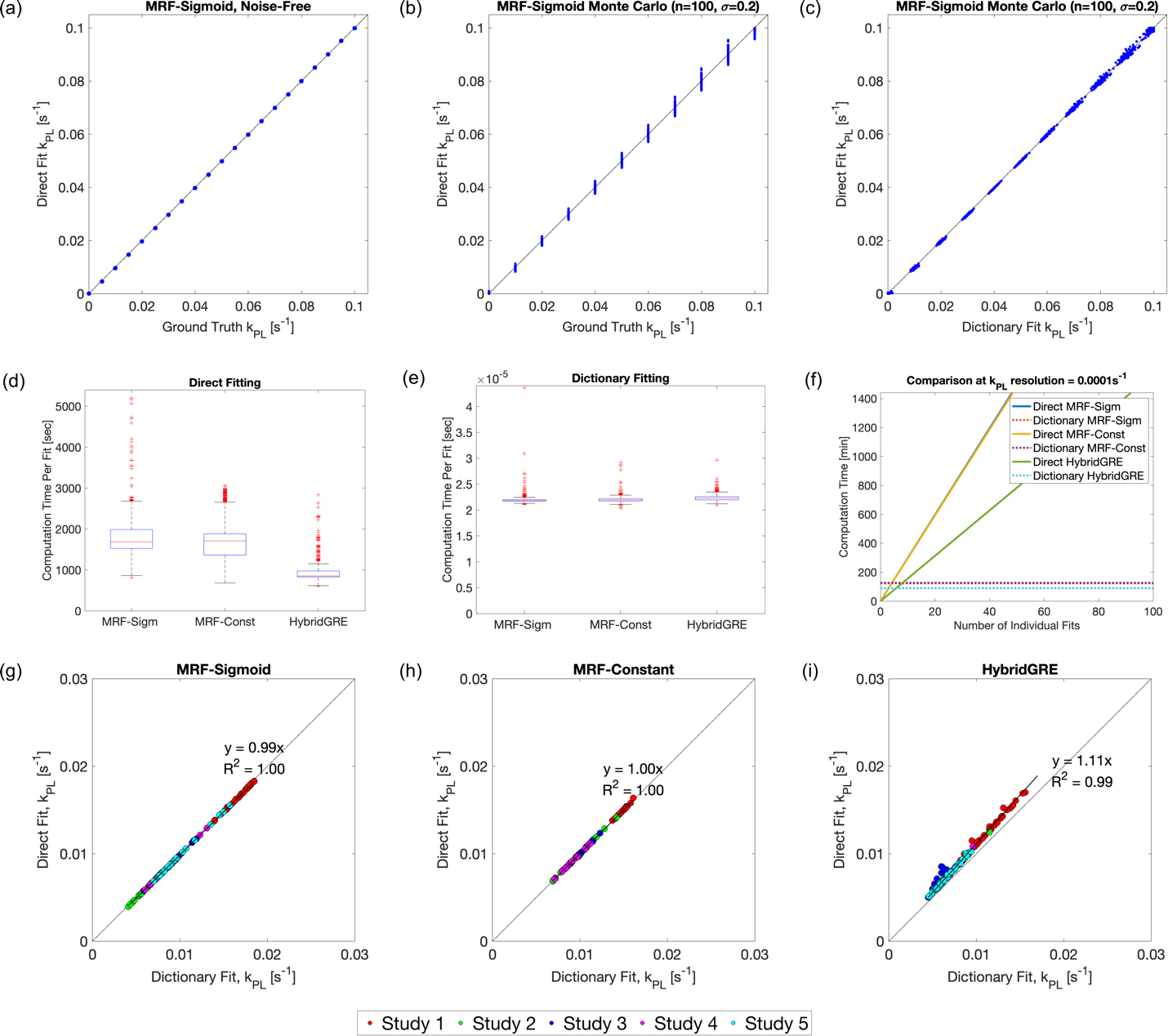


Figure S3: Kinetic rate estimation results from direct curve fitting using MATLAB lsqnonlin function for (a) MRF-Sigmoid under noise-free conditions and (b) under Monte Carlo evaluation (n=100, s=0.2). (c) Comparison of kinetic rate estimation of noisy data with direct curve fitting and dictionary-based template matching, showing average bias of -0.0002 s-1. Distribution of sequential fitting computation times are reported per single fit for (d) direct curve fitting and (e) dictionary-based template matching. (f) Expected combined computation time for dictionary-creation and template matching remains relatively constant with increasing numbers of estimations compared to direct curve fitting (requiring no upfront reference computations) which linearly increases. Voxel-wise kPL rate estimation for in vivo datasets fit via dictionary-based template matching and direct curve fitting directly compared via correlation plots for (g) MRF-Sigmoid, (h) MRF-Constant, and (i) HybridGRE experiments.


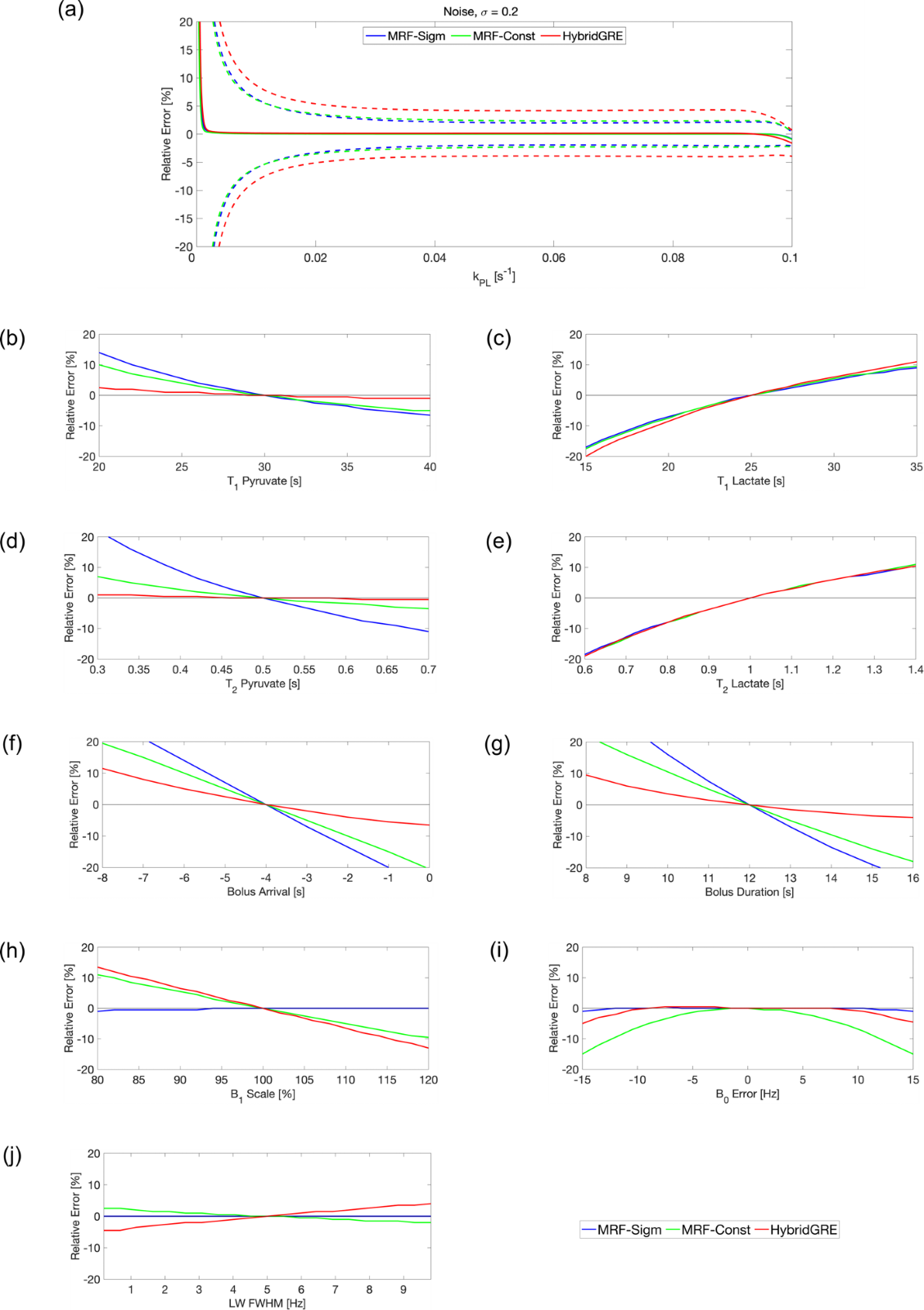


Figure S4: Monte Carlo simulation results across the full dictionary reported as error relative to the ground truth kPL value. The relative error between simulated and fit kPL is also reported for (b) pyruvate T1, (c) lactate T1, (d) pyruvate T2, (e) lactate T2, (f) bolus arrival relative to acquisition start, (g) bolus duration, (h) B1 relative scale, (i) B0 error and (j) LW full width half max. The simulated signals including sensitivity offsets were fit with the dictionary defined by the nominal sensitivity value for each experimental design independently for MRF-Sigmoid, MRF-Constant, and HybridGRE, respectively.
